# A case for personalised medicine in a 17 year old patient with obsessive compulsive disorder

**DOI:** 10.1192/j.eurpsy.2025.1109

**Published:** 2025-08-26

**Authors:** T. Bollain Muñoz, I. Oliveira Amat, R. Crespo Rubio, T. Gomez Escribano, J. Gomez Puebla, J. M. Martinez Avila, J. J. Carballo Belloso

**Affiliations:** 1 Psychiatry, HGUGM, Madrid, Spain

## Abstract

**Introduction:**

Pharmacogenomic testing is a cutting-edge precision medicine tool that analyzes genetic variations influencing drug metabolism. By assessing an individual’s unique genetic profile, this testing enables the personalization of treatment strategies, improving therapeutic outcomes, and enhancing patient care. Integrating pharmacogenomic testing into clinical practice holds great promise for improving the efficiency and effectiveness of mental health care delivery. In this case, a 17-year-old patient presented with a severe case of obsessive-compulsive disorder showed no response to treatment with sertraline (250mg). Sertraline is metabolized into N-desmethylsertraline through multiple pathways, including CYP3A4, CYP2C19, CYP2B6, and other CYP enzymes, with pharmacokinetic studies identifying CYP2C19 as the primary metabolic pathway.

**Objectives:**

The patient had a poor response to pharmacological treatments previosly used, our aim was to determine the possible involvement of patient specific responses to treatments based on his pharmacogenetic proffile.

**Methods:**

A blood sample was submitted for pharmacogenetic testing. This analysis includes genes involved in the metabolism of sertraline (CYP2C19, CYP3A4, and, to a lesser extent, CYP2B6 and CYP2D6) as well as other pharmacogenes associated with the metabolism and response to psychiatric medications, including HTR2A, OPRM1, COMT, and DRD2.

**Results:**

**Gene** | **Genotype** | **Inferred Phenotype**

- **CYP2C19** | *1/*1 | Normal Metabolizer
- **CYP2B6** | *1/*1 | Normal Metabolizer
- **CYP2D6** | *3/*4 | Poor Metabolizer
- **CYP3A4** | *1/*1 | Normal Metabolizer
- **OPRM1** | AA | Normal Genotype
- **HTR2A** (rs7997012 A>G) | GG | Normal Genotype
- **HTR2A** (rs6311 G>A) | GA | Heterozygous
- **DRD2A** (rs1799732 G>-) | GG | Normal Genotype
- **DRD2A** (rs1799978 A>G) | TT | Normal Genotype

**Conclusions:**

The patient did not exhibit clinically significant alterations in the metabolism of sertraline (CYP2C19, CYP3A4 and CYP2B6). However, the lack of response to treatment should be further investigated, factors such as potential drug interactions, and other variables including age, renal function, and liver function should be considered. In contrast, the patient has notable alterations in CYP2D6 and HTR2A, which could be important for guiding future treatment decisions. Variants in HTR2A can significantly influence a patient’s response to antidepressants, particularly selective serotonin reuptake inhibitors (SSRIs), specific polymorphisms in HTR2A, such as rs7997012 and rs6311 have been associated with differences in treatment outcomes, side effects, and remission rates. Has a CYP2D6 poor metabolizer, this patient may be at risk for higher drug levels and increased side effects when taking medications such as venlafaxine, fluoxetine, paroxetine (SSRIs), haloperidol, and risperidone.

**Disclosure of Interest:**

None Declared

